# ‘All My Animals Are Equal, but None Can Survive without the Horse’. The Contribution of Working Equids to the Livelihoods of Women across Six Communities in the Chimaltenango Region of Guatemala

**DOI:** 10.3390/ani11061509

**Published:** 2021-05-22

**Authors:** Molly A. Vasanthakumar, Melissa M. Upjohn, Tamlin L. Watson, Cathy M. Dwyer

**Affiliations:** 1 16 Penybont, Govilon, Abergavenny NP7 9RA, UK; 2 Dogs Trust, 17 Wakley Street, London EC1V 7RQ, UK; melissa.upjohn@dogstrust.org.uk; 3 The Donkey Sanctuary, Slade House Farm, Sidmouth, Devon EX10 0NU, UK; tamlin.watson@thedonkeysanctuary.org.uk; 4 Easter Bush Campus, Scotland’s Rural College (SRUC), Edinburgh EH25 9RG, UK; cathy.dwyer@sruc.ac.uk

**Keywords:** working equids, animal welfare, women, gender roles, Central America, extension services, education

## Abstract

**Simple Summary:**

Working equids are often absent from higher level policy interventions, and the global standard of their welfare is low. Understanding the social and cultural context of their contributions to human livelihoods generates evidence supporting the importance of their inclusion in livestock welfare programmes. Although there is increasing evidence globally that working equids contribute to women’s livelihoods and that women facilitate equid welfare, there is a well recognised gender gap in access to extension services. This study aims to investigate how working equids contribute to women’s livelihoods in six communities in Guatemala, using information from 34 face-to-face interviews. Results show that working equids support women’s livelihoods by generating income, saving time, feeding livestock and reducing domestic drudgery. Thirty-two women played a major role in the daily husbandry of working equids, and 31 expressed an interest in gaining more knowledge in equid care. This study explores the relationship between working equids and women in a local context, using the concept of ‘One Welfare’, and investigates the knowledge gaps that exist in the daily husbandry of horses, donkeys and mules. It emphasises the need for equid welfare organisations to understand women’s roles in their target communities and recognise what prevents women from accessing educational resources.

**Abstract:**

It is widely assumed that working equid husbandry is carried out by men, and women are often not recognised as facilitating equid welfare. The aim of this study is to investigate how working equids contribute to women’s livelihoods in six of the World Horse Welfare programme target communities in Guatemala and determine what roles women have in their care. Thirty-four face-to-face interviews were carried out and data were analysed using both quantitative and qualitative methods. This study found that working equids support women by reducing domestic drudgery, generating income, feeding livestock and saving time. Thirty-two women played a major role in the care of one or more equids, and overall, women did not feel that they knew enough about equid husbandry. Thirty-one women said they would attend training opportunities if the advertising was clear and they felt that women were able to join. This study recognises the contribution of working equids to women’s livelihoods, describes the roles women play in equid husbandry and addresses the discrepancies between women’s roles and their capacity to undertake these tasks. This emphasises the need for extension services to include and cater for women, improving equid welfare and their ability to continue supporting women’s livelihoods.

## 1. Introduction

There are approximately 116 million equids in the world [1], the majority of which are used as working animals in low- to middle-income countries (LMICs) to reduce domestic drudgery [2], improve people’s social welfare and provide direct and indirect income [3,4,5]. Because equids contribute to livelihoods in ways that are different to that of food producing animals, policy makers often fail to recognise their importance, and therefore, working equids tend to be absent in most livestock related initiatives and policies [4]. This leaves them vulnerable to low welfare standards as the inherent value of their health and welfare is neglected [5]. Policy makers have traditionally associated men with owning working equids, and livestock initiatives aimed at women have tended to focus on poultry, dairy cows and small ruminants [6,7]. However, there is evidence to show that the type of livestock women own and care for varies greatly between regions, meaning that often women are the owners and/or carers of horses, mules and donkeys [7]. In the ‘Invisible Helpers’ report published by Brooke in 2014, women were found to be the primary caregivers for working equids in communities in Kenya, India, Ethiopia and Pakistan. The report also found that despite their involvement, they have little to no access to education on equid health and welfare [4]. This can be seen across the world and in various sectors, with women in LMICs making up 43% of the work force [8] whilst having consistently low participation in extension services [9]. Women are underserved by extension services for various reasons: services are not adapted to their needs [10], their work and roles are devalued in society [11], extension agents primarily work with household decision makers who are often men [12], there is widespread female illiteracy [13], and women suffer particularly long working days both in the field and then at home [7]. For example, in India, women’s roles in agriculture are increasing, a theme referred to as the ‘feminisation’ of agriculture [14]. Whilst increased responsibility may be thought of as a mode of female empowerment [15], increased workload has been strongly related to indicators of poverty and has been instead referred to as the ‘feminisation of agrarian distress’ [16]. As women must divide their time between their various responsibilities, time trade-offs exist between activities [17]. The overall high time burden that women in low to middle income countries have as a result of their involvement in agriculture leads to time constraints. The implications of time constraints have variable impact between households due to factors such household income, ability to purchase food, household composition and work intensity [18]. An increase in women’s roles has no inherent relationship to the wider indicators of their social or economic empowerment [16]. To promote the upward mobility of rural women, their work and responsibilities must be recognised as value-generating rather than an extension of household work [19], and they must be offered the resources and knowledge needed to carry out those tasks [16].

The 1996 Guatemalan Peace Accords, created following 36 years of internal conflict, are widely recognised for their inclusion of women’s economic, political, and social rights, creating specific mechanisms to protect indigenous women [20]. Despite those advances, Guatemala is a male-dominated patriarchal society, with widespread inequalities. Indigenous communities are excluded in general and women specifically. Gender inequality gaps particularly exist in areas including family decision making, access to assets and resources and political and social participation. These gaps are greater for rural indigenous groups [21]. In Guatemala, there is a paradox between the relatively low unemployment rates (2% in 2019 [22]), with high levels of poverty, and consistent migration of men away from rural areas in search of employment [23]. As men migrate, it is suggested that agriculture in Guatemala, as in India [14], is beginning to ‘feminise’, as women begin to gain further agricultural roles. However, they still face challenges, not least of which are limited access to training and to credit and very low rates of land ownership, at 7.8% [24]. 

Gender roles are thought to be highly contextual with gender relations varying not only by individual characteristics but also by community features [25]. The Chimaltenango region has an economy mostly reliant on crop agriculture and, to a lesser degree, livestock [26]. Historically, it is the land of the Kaqchikel people, an indigenous Mayan group [27], and faced some of the worst violence during the armed conflict between 1981 and 1983 [28]. Similar to the wider culture in Guatemala [21], the region has a patriarchal society; traditionally, women receive a basic education, marry at approximately 17 years of age and work within their homes and on the family farm. (D. Rodriguez, personal communication, 30 March 2020). Research in the Chimaltenango region on the economic valuation of working equids demonstrates that working equids are particularly valuable to small producers. Equids are of major importance in coffee and corn crop transportation, and if working equids were lost, families would be impacted through the loss of their productive assets [29]. However, these data outline the economic importance of working equids in productive processes and do not consider their value in providing other forms of capital or focus on gender related influences, which are the subject of this study.

The majority of services offered by working equid welfare organisations are intended primarily to serve the health and welfare needs of the animal [30]. However, we are becoming increasingly aware of the contributions that healthy, productive equids have to human livelihoods [4,30,31,32], drawing on the concept of ‘One Welfare’. ‘One Welfare’ recognises the connection between animal welfare, human wellbeing and the environment to generate solutions designed to benefit the welfare of all species [33]. The present study builds on this concept and investigates women’s roles in working equid care across six communities in Guatemala and links those roles to the welfare needs of working equids. This study aims to investigate (1) how working equids contribute to women’s livelihoods in the Chimaltenango region, (2) the roles women have in caring for their equids and their capacity to undertake these tasks, (3) the opportunities women have to acquire new knowledge about their equids, (4) whether women find existing training programmes helpful and accessible and (5) the areas of equid care on which they would like more information.

## 2. Materials and Methods

### 2.1. Study Site

The study consisted of interviews across six communities in Guatemala: Las Colmenas, La Soledad, Las Lomas, El Campamento, Mancheren and San Rafael. These communities are situated approximately 75 km west of Guatemala City in the mountainous Chimaltenango region. La Soledad, El Campamento and San Rafael are in close proximity to the volcano complex La Horqueta and were devastated by the 2018 eruption of Volcán Fuego.

World Horse Welfare and a local charity *Servicios de Apoyo en Bienestar Equino* (SABE) have worked in Las Colmenas, Las Lomas and Mancheren since 2013 and have well established Equid Welfare Networks (EWN) in place. An EWN consists of a saddler, farrier and a Community Based Equine Advisor (CBEA). The CBEAs run training projects teaching equid owners about basic care and management, and whilst they are supported with training and a revolving fund, they are not permanent employees of either World Horse Welfare or SABE. If their assistance is needed with a community visit, then they may occasionally be hired by SABE. In 2018, World Horse Welfare and SABE began collecting data about working equid populations and building relationships in San Rafael, La Soledad and El Campamento. They offer promotional services such as free veterinary and farriery advice, but owners have not yet been offered any training opportunities and there are no established CBEAs.

### 2.2. Data Collection

The present study consisted of 34 face-to-face structured interviews, obtaining descriptive insights from participants into the five study aims. Access was kindly granted to a questionnaire used in the earlier Invisible Helpers project [4]. The format was restructured around the present study’s five aims, questions related to vulnerability and resilience were removed, introductory background questions were added and it was adjusted to fit individual interviews rather than focus group discussions (see questionnaire in supplementary materials). Individual interviews were deemed most appropriate for the present study as, due to logistical constraints, it was not possible to obtain the services of a translator experienced in the format of focus group discussions.

Interviews were conducted between 24 September 2018 and 12 October 2018. The questionnaire was translated into Spanish by a World Horse Welfare employee prior to the study commencing and was then checked by SABE’s veterinary intern who was the translator for the present study. As the questionnaire had been successfully used in the Invisible Helpers research, pretesting prior to data collection comprised a mock interview with an employee of SABE as the participant. The translator asked the questions to the participant and translated the responses into English for MV to transcribe. This enabled the translator and MV to practice carrying out a mock interview and to check whether any changes to wording or format were required—no changes were identified as needing to be made. The interview team was made up of a translator (female) and a transcriber (MV–female). The CBEA (male) was also present for those communities with established EWNs. For communities without a CBEA, the interview team consisted solely of MV and the translator. For five interviews on a single day, the translation was conducted by one of SABE’s equine vets (female) rather than the intern. This was necessary on that day due to staff availability. Women were eligible to participate if a member of their household owned one or more working equids.

Each interview began with a short introduction from the translator, introducing herself as a SABE employee facilitating MV, an independent researcher, in carrying out a research project with World Horse Welfare, a partner organization of SABE. Whilst it was assumed that many women would already know the CBEA as a member of their community, they were introduced anyway and their work with SABE was stated. It was recognised that introducing the interview team in this way may have influenced participants’ answers, especially when discussing the importance of working equids. However, SABE and World Horse Welfare have developed a good rapport among the communities, even within the three communities that did not have CBEAs, so it was felt that women may feel more comfortable discussing their experiences of working equids with organisations that they had heard of. The questions were asked in Spanish; answers were translated back to English and written down by MV. It is acknowledged that this method of contemporaneous translating and transcribing may cause discrepancies between what the women said and what was transcribed. Interviews were not recorded because of concerns that women may have been reluctant to participate if they were being recorded, as well as resource constraints of employing a bilingual interpreter for the task of transcribing and translating interviews at a later date. The questionnaire format was followed for each interview, but the order of questions was dependent on the direction and flow of the conversation. Whilst not every question was asked to every participant, the five study aims were discussed in each interview. There were opportunities for participants to explore certain areas in more depth if they wished to, however, the majority of questions had short responses. If participants expanded their answers beyond the initial question or wished to provide anecdotes of certain events, then the translator could ask their own questions to facilitate the conversation. If a response unintentionally answered a question from later in the questionnaire, that question was not asked again.

CBEAs are an important part of the communities; their knowledge of the area and the people was essential to guide recruitment of women they felt would have the time and inclination to be interviewed. Prior to data collection, the CBEAs were given information about the aims of the research; they then gave advice on women who might like to participate. Women had never been specifically approached by SABE before, so whilst the CBEAs’ advice on who to interview is recognised as a bias, it was necessary due to the lack of confidence women had when it came to sharing their experiences. In El Campamento, La Soledad and San Rafael, data were collected by the researcher (MV) and translator during SABE’s promotional days. These promotional days included members of the community bringing their equids for veterinary advice and farrier checks in a communal area of the village. For these communities, a convenience sample was used as there were no CBEAs to offer advice on participant selection. It has been recognised that interviewing participants whilst offering a promotional service creates a possible bias and an issue surrounding informed consent [34]. To mitigate this, all interviews were carried out in the women’s homes or farms, rather than in the area where the vets and farriers were working. This was aimed at reducing the idea that women must participate in order to receive free services or advice.

For the small number of interviews (approximately five) carried out with men other than the CBEA present, some women were initially keen for men to answer questions on their behalf. All responses used in the study were those that came from women themselves, without input from any men present, although it cannot be ruled out that the presence of men may have influenced women’s responses. All interviews were carried out in the women’s houses or in their fields. As the majority of women work in their homes or fields, it was assumed that carrying out interviews in those settings would cause them minimal disruption.

### 2.3. Data Analysis

Each participant was allocated a unique key informant (KI) number to ensure that data were pseudoanonymised. Data had already been transcribed into the written form during the interview stage but transcripts were copied out by MV into a clearer format for data analysis, taking care to ensure that the meaning of the text was not altered and no changes to grammar or syntax were made. A thematic approach to data analysis was adopted, as described by Braun and Clarke [35], using simultaneous inductive and deductive methods. Data analysis was carried out by MV, and whilst there was prior knowledge of the data and initial analytic interests from the interview stage, the first stage of data analysis involved reading transcripts several times over. Initial codes were then produced systematically from the entire data set, one questionnaire at a time. This was done manually by using highlighters and post-it notes to identify as many potential patterns as possible. It is recognised that whilst the entire data set was coded, the data were approached with an understanding that subsequent themes would be more ‘theory driven’ with links to the five study aims described in the introduction. The various codes from each questionnaire were then sorted into themes. Narratives were then created for each theme from the collated data extracts. Sub-themes were used to enhance the narrative by providing a more in-depth insight into the participants’ experiences of working equid care. The main themes and subthemes that emerged from the study form the structure of the results section. As interviews had been transcribed in real time, particular quotations were pulled out to enhance the story. These have been included in the results along with the participant’s KI number and information on whether there was an established EWN in the community to demonstrate the level of intervention that the community had received from World Horse Welfare and SABE. 

### 2.4. Institutional Review Board Statement

All subjects gave their informed consent for inclusion before they participated in the study. The study was conducted in accordance with the Declaration of Helsinki, and the protocol was approved by the University of Edinburgh, Royal (Dick) School of Veterinary Studies Human Ethical Review Committee.

## 3. Results

### 3.1. Background Information on Participants

The median age of participants was 37 years old (range 18 to 76). Thirty-two women lived with their parents or husbands, and two women lived in women-headed households. A household was defined as a ‘women-headed household’ if the women said they did not live with a husband. There were 34 households in total, and all relied heavily on agriculture for income. Four had additional income from other employment, such as factory work (n = 1) or employment by SABE (n = 3). The daily roles of all women were to work within their home, however, six women also spent time working with their husbands in the fields, three ran their own businesses and one worked as a local midwife (S1). Out of 33 women, eight did not have any formal education, 21 had received some primary school education and four had continued until secondary school.

**Figure 1:**
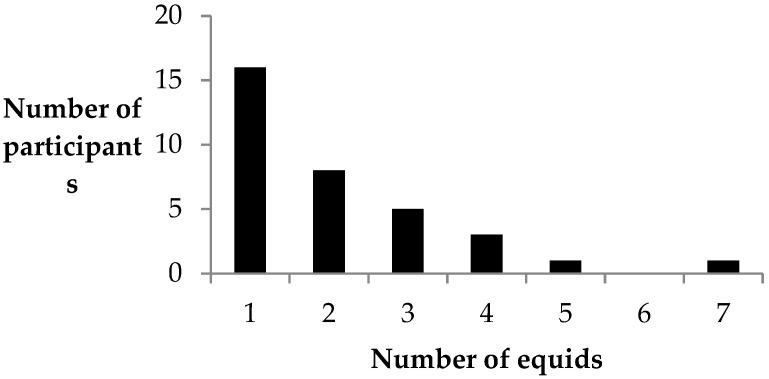
The number of equids reported to be owned/cared for by 34 women in the Chimaltenango region of Guatemala.

Women were most likely to have a single equid (n = 16) owned by their household but some had up to seven (Figure 1). All women owned/cared for a horse but two women also had a mule or donkey. Other owned livestock included goats, pigs, chickens and cattle. 

### 3.2. Themes and Subthemes

The themes and subthemes that emerged from interviews with the study participants are described in Table 1.

### 3.3. Working Equids Are Important to Women’s Livelihoods

When asked to rank their livestock, 21% (n = 7) of women felt that their equids were the most important animals they owned. One woman said, “Horses give life”(KI 8—established EWN), and another said, “The horse is the only animal that can carry their own food” (KI 34—no established EWN). Half (n = 17) of women said that all their animals were equally important: “All my animals are equal, but none can survive without the horse” (KI 22—established EWN). The remaining 29% (n = 10) who ranked their equids lower than other participants still acknowledged that equids were integral to the wellbeing of their other livestock. All participants owned food-producing animals, from keeping a few chickens, to running a cattle fattening farm. Almost all (n = 33) women felt that their equids contributed to food production by transporting firewood for cooking and food and water for other livestock

### 3.4. Income Generation

Transporting wood, fodder and crops were cited in every community as ways in which working equids generated income. Other methods of income generation included hiring for a fee, tourism and breeding (Table 2). One women stated that without an equid, her family would be unable to save money. However, it was reported that many households own working equids but still struggle financially: “Many people have a horse, but still no one has much money” (KI 17—established EWN). One woman discussed the fact that equids did not necessarily remove a household’s struggles: “Everyone here has a horse, but everyone still has needs” (KI 12—established EWN). She felt that although equids gave opportunities for greater income generation, it was dependent on how they were used. She said that working equids generate income primarily from transporting firewood for sale; therefore, if the family is not in the firewood business, their equids have less ability to make money. This theme of income generation being dependent on use was reiterated by 71% (n = 24) of women. One woman also said: “Households with equids do not always have more money because many people are just bad with money” (KI 1—established EWN). It was also noted by one participant that the capacity to make an income from agriculture had reduced in her community, and men were now able to make more money by gong to work in the nearby cities and towns. This meant that many working equids were being used less and therefore making less money for their households. All women stated that equids were not used to obtain loans or take out credit, but during discussion it was noted that equids gave them some security because they could be sold quickly if they were desperately in need of money. However, it was apparent that this was only a short-term contingency measure, as the financial return from selling an equid was considerably less than the cost of purchasing a new one.

### 3.5. Household Chores

All women (n = 34) expressed the importance of equids in reducing domestric drudgery, by transporting firewood and/or water, tasks that require manual effort: “The work of horses cannot be done by man” (KI 18—established EWN). Whilst it is men who mostly use equids for transporting goods for sale, women said that without equids, the time consuming and physically exhausting job of carrying wood and water for the household would often pass to them. One woman said: “If my horse was sick, I would have to carry the wood myself, and this takes all day” (KI 11—established EWN). Another said: “Working without a horse is backbreaking for me” (KI 12—established EWN). Having an equid to help with household chores saves time for women. Women reported that the time equids save can be used to do other chores (n = 29), rest (n = 6), look after children (n = 8), look after other livestock (n = 2) and other income generating activities (n = 3). Whilst discussing time saving, one woman said: “My horse means I have more time to tend to my cows and sell cheese in different communities” (KI 23—established EWN).

Five women stated that although the overall physical workload of their work was reduced, caring for equids actually gave them more chores: “Our horse gives us more chores to do, but they are much less physical than our chores if we didn’t have a horse.”(KI 13—established EWN). Two of the five women who felt that caring for equids increased their daily tasks said it was because their equids were too young to work. Owning young equids mean that women carry out the daily husbandry tasks to care for the equid but also have to do the jobs that would normally be carried out by a more mature animal, such as carrying firewood. One of the two women noted that young equids are more difficult to care for than older ones because they are still learning and that their behaviour is more difficult. However, they both noted that this would change once their equids were older and able to take on a greater workload.

### 3.6. The Impact on Women When Equids Cannot Work

All women said that their lives would be impacted to varying degrees if their equid died or was unable to work. Many had short-term measures in place to reduce the impact on their livelihoods if their equid could not work: “We have an extra supply of wood in case our horse cannot work” (KI 28—established EWN). Other strategies described by women included: hiring a truck to transport wood and crops, purchasing firewood locally for household use, selling firewood to people with vehicles so that they can collect and carying firewood themselves. If the household owned more than one equid the workload could be carried out by another: “When one horse is sick, the work increases for the others” (KI 3—established EWN). Owning multiple equids was also noted by one woman as an advantage as you could ensure that they were given rest days. An older woman with only one equid expressed her concern that they would not be able to manage if their equid could not work: “Without a horse we will all suffer, as my husband is too old to work without one” (KI 8—established EWN). Women discussed the fact that they did not have the money or resources to cope should their equid be unable to work for extended periods of time, or if their equid died.

Of the 34 women, 29 women said they would use local medicinal treaments before veterinary interventions if their equid was unwell; this is because they are easy to get hold of. The remainder (n = 5) said that they would call a vet first as they did not have any knowledge on medicinal plants. Women mentioned specific treatments for ailments including: treating colic with garlic, treating herbicide poisoning with beer and applying vinegar to their backs to make them less likely to develop rubs from tack. In order for equids to receive veterinary treatment, families would be prepared to borrow money from friends and family: “I will borrow money from neighbours to pay for the horse’s vet bills” (KI 16—established EWN). Out of 34 women, 33 said that they would have to save up to buy another equid if theirs died, placing more strain on families: “If our horse dies, we will fight to buy another” (KI 29—established EWN).

### 3.7. Caring for Working Equids

Within the communities visited, 32 women were the primary caregivers for all livestock including working equids. Two women said they had no role in caring for their family’s equids due to a preference for chickens and cattle and limited amounts of time. The remaining 32 women said that their basic roles consisted of feeding and watering equids and cleaning the area where they were tethered. Six women also groomed their equid, and four said that they like to give them a bath when the weather was warm. One woman said that she liked to feed her equid bran when he was tired from working. The two women-headed households carried out all aspects of equid care.

Only 15% (n = 5) of women were actively involved in using their equids, one of which was from a woman-headed household. Women reported that their main constraints in using their equids were fear of the equid (n = 18), gender-specific roles and commitments (n = 18) and time (n = 4). The eighteen women who were scared of handling their equids said they had difficult temperaments and they were often afraid of getting hurt. Other women discussed the fact that horses were not friendly towards people, could be bad tempered: “Horses get angry!” (KI 3—established EWN), and that they would only use an equid if it had a good temperament. Fear over being kicked when handling their equid’s hooves and legs was commonly reported amongst participants. One woman said that the fact that men were often working away from home meant that equids were left without work because women were too afraid to use their equids in the way that the men did. She explained that her fear stopped her from being able to use her equid: “My husband is away working, but I cannot use my horse as I am afraid to work it alone” (KI 15—established EWN). Another woman, whose husband also worked away, said that her equid was beginning to get used to her but that she was still too afraid to use it. Three participants mentioned that women have less confidence to handle equids as they get older. They said that younger girls will often ride their equids without fear, but that once they are older and have a family, they get scared. One woman stated that she used to use her family equid until she had a caesarian section with her first child, and then she was afraid of being hurt.

### 3.8. Decision Making

When asked who makes decisions on how equids are used, how the income made from equids is spent, whether equids are lent to other families and how much money to spend on treating them if they are sick or lame, it became apparent that decision making was carried out mostly by equid owners. Women explained that they viewed an ‘owner’ as someone who personally used the animal for work, a role that is often carried out by men. Only participants from woman-headed households chose how their equids were used, and of these two women, only one directly used her equid. Women said that owners are the people who attend the training opportunities offered by SABE.

### 3.9. The Social Benefits of Working Equids

Only 15% (n = 5) of women said that they used their equids to socialise or to visit family and friends, one of whom lived within a woman-headed household. The remainder (n = 29) said that it is only men who use their equids for social functions: “We have two horses but when we go to visit family, I must walk whilst my husband and children ride” (KI 5—established EWN). Of the 34 women, 14 said that on occasions their equids are loaned to friends and family, but as it is men who make decisions regarding the loaning of equids, it is men who receive the social benefits such as respect and gratitude from community members. However, when 30 women were asked whether owning/caring for equids made them more or less respected in their community, 17 said they were more respected, 11 said that it did not make a difference and two said that they did not know. However, it became apparent that respect was mostly obtained from being seen to handle equids rather than simply owning one. One woman stated: “You are respected if you are seen to use your horse, even putting on a saddle is brave” (KI 32—established EWN); another said: “There is jealousy amongst women who don’t own a horse” (KI 18—established EWN). One participant described people stopping and staring if a woman was seen using a equid, and another said that they would admire a woman who could handle her equid.

### 3.10. Knowledge on Equid Care, Skills and Capacity Building

When asked how women had learnt about caring for equids, 25 women said that they gained knowledge from their husbands and fathers, and the remainder (n = 9) said that their knowledge on equid care had come from growing up around livestock. Of the 34 women interviewed, 19 felt that they did not have enough knowledge to care for their equid properly (Table 3). Of the 28 women living in communities with established equid welfare networks, 22 said that their personal level of knowledge had increased since the projects were started. In the three communities where SABE have offered extension services, 24 out of 28 women said their husbands or fathers attend the training events. The remainder (n = 4) said that no one in their family attended because they were not aware of them or did not have time. The reasons that women did not attend were that they did not have time (n = 20), they were not aware of the opportunities (n = 16), and that they thought training events were primarily meant for men (n = 7).

**Figure 2:**
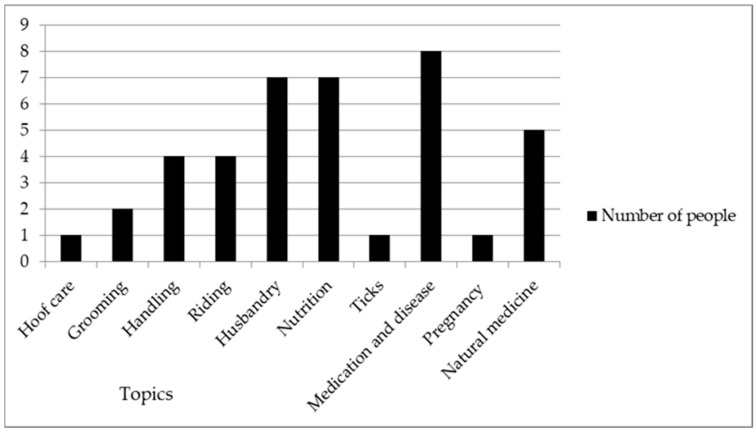
Equid husbandry topics that women in Chimaltenango, Guatemala, expressed an interest in learning about if offered access to training opportunities.

When asked if women would attend future training opportunities, 31 women said that they would like to. However, it was apparent that there were barriers that would need to be addressed before women could attend. Women suggested that events would need to be advertised clearly as often women did not know they were being held, and it would need to be evident that women could attend. Some would need permission from their fathers or husbands. With regards to time, women suggested that local events in their village would be the most accessible, and there would need to be activities put on for the children to reduce the concerns around childcare. Fifteen said that they would prefer future training to be just for women, two said women and children, and fourteen women said they should be for everyone. Of the 31 women who wished to attend training opportunities, the most popular topics to learn about were: medications and disease, general husbandry, nutrition and natural medicine (Figure 2).

## 4. Discussion

Results from the present study show that working equids in Chimaltenango are important to women’s livelihoods. They reduce domestic drudgery, generate direct income and save time. Time saved may increase a family’s daily income and provides women with more time to care for their homes, other animals and children and to rest. Working equids influence food production by helping women care for other livestock through transportation of fodder and water. The present study in Guatemala has shown that women in this region are the primary caregivers of working equids. Similar findings were apparent in communities in India, Pakistan, Ethiopia and Kenya according to the Invisible Helpers report [4].

Women in the present study felt that their equid’s ability to generate income was dependent on the way that they were used, supporting the results of a study measuring the economic valuation of working equids within Chimaltenango [29]. The study found that although economic evaluation indicators were higher across all systems using equids, the added value provided by equids differed based on the agricultural system, the distance of transportation and whether the produce was for family consumption or commercial sale [29]. In the present study, results suggested that whilst caring for working equids increases women’s daily chores, this is offset by a reduction in the heavy domestic work generated from collecting and carrying firewood and crops, tasks that would often be assigned to women. As the primary caregivers, some women stated that they enjoy grooming and bathing their equids, practices that can be considered additional to the essential daily tasks such as providing feed and water. Women often reported a lack of time due to their many family and household responsibilities, so carrying out non-essential tasks to improve their equids wellbeing may demonstrate the appreciation that women have for their equids, or a level of enjoyment in caring for equids.

Whilst data from the Invisible Helpers report showed that women were the direct users of working equids [4], most women in the present study said that they did not use their equid themselves and that their roles were restricted to husbandry tasks. This decision was mostly driven by fear of equids, lack of knowledge and/or skills in handling and traditional gender roles. Research suggests that the animal husbandry practices assigned to men and women across the livestock sector are country and community specific [36,37]. Rural poverty in Guatemala increased from 74.5% to 76.1% between 2000 and 2014 [38] and has been recognised as one of the drivers of rural men migrating away from rural areas to find employment or transitioning from agricultural to non-agricultural sectors [39,40]. This feminisation of agriculture creates the potential for rural women to face an increase in their workload and a greater demand for them to use or handle equids. This was also noted in the brick kilns of northern India, where the lack of visibility of women’s roles in donkey husbandry created challenges during the off-season when male workers had to find employment elsewhere, leaving women to take over donkey management without the resources or confidence to do so [41]. The feminisation of agricultural roles could also create greater pressure on women’s time resources and may build a further barrier to them being able to access conventional training opportunities offered by equid welfare organisations. Women in the present study stated that fear and gender roles were barriers that prevented them from wanting to or being able to use their equids. Women said that their fear of equids increased with age, especially after starting a family. This suggests that women’s fear could originate from gender roles creating a perception that working equids are dangerous and therefore best handled or used by men.

As women did not generally use their equids, they did not view themselves as ‘owners’ of equids and therefore often weren’t able to access the social and educational benefits of ownership or have involvement in decision making regarding their equids.The demographic of working equid ‘owners’ has been reflected in studies globally [30,42,43], with recognised differences between predominantly female ‘caregivers’ and male ‘owners’ [4,44]. This theme is mirrored in agriculture, where women are often described as ‘caregivers’ to food-producing livestock [45]. The idea of gender and animal ownership within the present study supports the findings of Mudege et al. [46], where men were perceived to be household heads with women as illiterate or poorly educated carers and helpers. This gender bias denies women access to information and extension services, as well as devaluing their roles and responsibilities. Gaining a better understanding of how gender roles surrounding working equid husbandry are assigned could identify methods to support women in carrying out equid husbandry tasks safely and confidently in the absence of men, should they have to, or wish to. It could also provide women with opportunities to obtain the respect given to women who are seen to use and handle their equids, from members of the community.

Whilst results from the present study showed that women often have contingency plans to put in place in the event of their equid being able to work, these are only short-term measures. The high cost of purchasing a new equid means that many families would struggle to find the money to purchase a new equid if theirs died. It is recognised that the negative impacts experienced when an equid is unable to work means that owners are often forced to continue working their equids even when they are suffering from painful and debilitating injuries [41]. Women in the present study stated that if their equids became unwell, they would prefer to use local medicinal treatments before getting veterinary help. Traditional medical treatments are often disregarded and devalued as having no impact on the condition being treated [47]. Terms to describe traditional medicine such as ‘alternative’ suggests a power differential to more conventional interventions [48]. Recognising the importance of medicinal treatments ensures that women in rural areas who may struggle to access veterinary services or pharmacies can treat their animals effectively on locally available and affordable resources. Prompt and effective treatment of conditions can mitigate equid welfare concerns and reduce the impact that ill health has on women’s livelihoods.

The vast majority of the women in the present study said that they did not have enough knowledge to care for their equid and wished to learn more. Currently women’s attendance at local training groups is limited; these gender disparities in access to extension services exist throughout LMICs [7]. Extension services are often delivered based on land or livestock ownership and therefore female ‘caregivers’ are more likely to be neglected in access to support [45]. From the results of the present study, it is clear that women were not always aware of existing training opportunities or thought that they were intended solely for men. Many women need permission from their husbands or fathers to attend, a theme common in patriarchal societies where gender norms restrict women from participating equally in decision making [49]. Gender inequality is common in Guatemala, and women have the lowest literacy rate in Central America at 76% [50]. Whilst they have equal rights to men to own land and take out bank loans, in practice their freedom is restricted by cultural practices [51]. The present study shows existing training programmes offered by World Horse Welfare impact women through indirect knowledge transfer from men. This pattern has also been seen in Pakistan where women obtained information on husbandry from their husbands, who were involved in working equid groups [4]. This emphasises the need to engage with men to discuss the importance of access to equid welfare programmes for women and to better appreciate the local gender norms that shape women’s roles in society.

Whilst working equids can technically be defined as livestock, they are often not considered as such because they do not produce food of animal origin. As a result, equids are rarely included in livestock-related policies and guidelines [5]. In 2017, Guatemala’s Ministry of Agriculture, Livestock and Food approved a new animal welfare law to establish protection standards [52]. However, this law is not currently species specific and working equids do not have an individual law protecting them and their owners in Guatemala (D. Rodriguez, personal communication, 4th December 2018), despite the fact that women expressed the importance of good equid health to their livelihoods. If equids were unable to work for extended periods of time, women stated that the workload would increase for family members and other equids. Studies from across the world have identified the most common ailments affecting working equids, listing conditions such as lameness, colic, respiratory disease, epizootic lymphangitis and wounds [53,54,55]. There is evidence to show that equids in communities that have been offered services from welfare organisations are significantly healthier and more productive compared to communities that had not [30]. However, interventions that focus on the provision of services or bringing about a change in attitudes do not necessarily lead to long-term behavioral change [56]. Whilst knowledge transfer is a necessary component, it is not sufficient to facilitate changes in behaviour or mitigate the health and welfare conditions that working equids face. The COM-B framework describes a three factor model, whereby behaviour change is influenced by capability (both knowledge, emotional and physical capacity to engage), opportunity (acknowledgement of external factors, removal of barriers) and motivation (innate and social norms that direct behaviour) [57]. All three aspects need to be considered when reviewing a targeted change in behaviour, and community discussions regarding how best to facilitate all three are essential for development of sustainable interventions. Approaches that offer a long-term commitment to transformative change towards gender equity and equid welfare need to consider the socioeconomic and political systems in which equids and women live and put forward capacity building programmes that combine training with non-training strategies. It is important to factor in the likely positive and negative consequences of such interventions to ensure that women are not left worse off due to societal backlashes.

Using the concept of ‘One Welfare’, a healthy equid has the ability to support women’s livelihoods by reducing domestic drudgery, saving time and providing household income and social benefits. Helping women safeguard their livestock assets has been described by The Food and Agriculture Organisation of the United Nations as a pathway out of poverty [7]. In this context, safeguarding describes providing women with improved opportunities to care for their equids with interventions which can facilitate behaviour change targeting improved equine welfare, thereby simultaneously improving the welfare of women and addressing gender inequalities.

This research does not claim to be comprehensive, and the uneven sample size across the six communities is recognised as a limitation. In two communities, only one woman was interviewed. Whilst the communities are relatively socially homogenous in terms of ethnicity and reliance on agriculture to support the local economy, it is acknowledged that further data collection would give a greater depth of understanding of women’s narratives within these communities. Logistical constraints on SABE’s promotional days restricted the number of interviews which could be conducted. Those interviews were conducted in communities without an equid welfare network, and whilst the numbers of interviews from those communities were low, differences in exposure to equid welfare education may have influenced the responses compared to those from communities with longer established relationships with SABE. Another limitation was that for five interviews one of SABE’s equine vets carried out the translations. This may have influenced the overall consistency and accuracy. Some women were more hesitant than others when questioned about their lives and experiences, which made them reticent to answer without further encouragement. Therefore, some answers may not be a true representation of women’s opinions.

## 5. Conclusions

This study highlights the benefits working equids provide to women and their families in Guatemala. Women provide significant contributions to the husbandry of working equids, therefore, the lack of inclusion of women in policy and extension services may impact on working equid welfare. Further research is needed to identify social norms that affect women’s existing and desired interactions with equids, factors that influence their ability and interest in engaging with equid-related training, and the optimum structure of programmes for their daily commitments and other responsibilities, which facilitate human behaviour change targeting improved equine welfare.

## Figures and Tables

**Table 1:** The main themes and subthemes that emerged from interviews with 34 women in the Chimaltenango region of Guatemala regarding working equids.

Main Themes	Subtheme
Working equids are important to women’s livelihoods.	-working equids support food production.
Income generation.	-income generation is dependent on use. -migration of men impacts the ability of equids to generate income. -working equids can be sold in times of financial need.
Household chores.	-working equids reduce the physical workload of household chores. -working equids can save time for women. -working equids can generate more chores for women.
The impact on women when equids cannot work.	-women have short-term measures to cope if their equids cannot work. -women prefer to use local medicinal treatment for their equids.
Caring for working equids.	-women are the primary caregivers of working equids. -women do not use their equids. -fear and gender roles prevent women from using or handling equids.
Decision making.	-decision making is often carried out by owners, a role often assigned to men.
Social benefits.	-women gain respect in their community for handling working equids.
Knowledge on equid care, skills and capacity building.	-knowledge on equid care flows from male family members to women. -women would like to gain more knowledge on caring for equids. -there are barriers preventing women from attending training opportunities.

**Table 2:** Tasks that women in Chimaltenango, Guatemala, report equids are used for.

TaskTitle	San Rafael	El Campamento	La Soledad	Las Lomas	Las Colmenas	Mancheren
Transporting wood	✓	✓	✓	✓	✓	✓
Transporting fodder	✓	✓		✓	✓	✓
Transporting crops	✓	✓		✓	✓	✓
Transporting water		✓				
Hiring for a fee				✓		
Sale of foals				✓		
Transporting people					✓	
Tourism		✓				
Pleasure rides forthe family				✓		✓

**Table 3:** A case study discussing the gaps in women’s knowledge on caring for their equids in the Chimaltenango region of Guatemala.

Community	Case Study
4	One woman told a story where she fed her horse a type of fodder that she had not seen before, but assumed that it would be suitable for the horse to eat. Shortly after, the horse started choking. She ran to her parents and she was able to get some advice on what to do, and thankfully, her horse recovered. She expressed the importance of women learning about nutrition, so that incidences like this did not occur and the horses were able to be as healthy as possible.

## Data Availability

Background and demographic data have been pseudoanonymised and made available within the Supplementary Materials. The full transcripts are not publicly available as participants did not explicitly give consent for their release; however, they are available on request from the corresponding author.

## Supplementary Materials

The following are available online at https://www.mdpi.com/article/10.3390/ani11061509/s1, Table S1: Demographic and livelihood information of participants.
