# Supplementary material for: ‘All My Animals Are Equal, but None Can Survive without the Horse’. The Contribution of Working Equids to the Livelihoods of Women across Six Communities in the Chimaltenango Region of Guatemala
Source: Animals (Basel). 2021 May 22;11(6):1509. doi: 10.3390/ani11061509 (PMC8224632; doi:10.3390/ani11061509)
Supplement: Supplementary file 1 [file animals-11-01509-s001.zip › animals-1187186-suppl.pdf]

Supplementary Material

Table S1. Demographic and livelihood information of participants.

KI	Equid Welfare Network?	Age category	Who do you live with?	Main source of income	How is your time spent?	Other livestock owned?
1	Yes	40-49	Husband and children	Wood	Home	Chickens, cattle
2	Yes	40-49	Husband and children	Wood, livestock	Home	Chickens
3	Yes	30-39	Husband and children	Wood	Home	Chickens
4	Yes	20-29	Husband and children	Animal feed factory	Home	Cows, goats
5	Yes	20-29	Husband and children	Farrier	Home	Pigs
6	Yes	50-59	Husband, children, granddaughter	Farmer	Midwife, home, fields	Chickens, cattle, pigs
7	Yes	20-29	Husband children	Farmer	Home	Pigs, chickens
8	Yes	50-59	Husband and children	Farmer	Home	Chickens
9	Yes	60-69	Husband and children	Farmer	Home	Cattle, chickens
10	Yes	40-49	Husband and children	Farmer	Home and fields	Chickens, goats, pigs
11	Yes	20-29	Parents	Farmer	Home	Chickens, pigs
12	Yes	70-79	Husband and children	Wood and crops	Home	Chickens
13	Yes	40-49	Husband and children	Livestock, farrier, wood	Home and fields	Chickens, cattle, pigs
14	Yes	40-49	Husband, children, mother-in-law	Vegetables and livestock	Works on fields and at home	Chickens, pigs, cattle
15	Yes	30-39	Husband and children	Vegetables, livestock, factory	Home	Chickens, pigs
16	Yes	40-49	Husband and children	Vegetables, livestock	Home	Chickens, cattle
17	Yes	50-59	Husband and children	Vegetables	Home	Chickens, cattle
18	Yes	30-39	Husband and children	Vegetables, Livestock	Home and shop	Chickens, pigs, cattle
19	Yes	<19	Parents	Livestock	Home	Chickens, pigs, cattle
20	Yes	40-49	Children	Vegetables, livestock	Home	Cattle, pigs
21	Yes	20-29	Parents	Vegetables	Home	Cattle
22	Yes	60-69	Husband	Vegetables, wood	Home	Cattle, chickens
23	Yes	40-49	Children and grandchildren	Cheese	Cheese	Chickens, pigs, cattle
24	No	<19	Husband	Peaches, salad crops	Home	Chickens, pigs
25	No	<19	Husband	Peaches, salad crops	Home	Chickens, pigs

26	No	<19	Husband	Peaches, salad	Home	Chickens, pigs
27	No	<19	Father	Peaches, salad	Home	Chickens, pigs
28	Yes	30-39	Husband and children	Crops	Home	Chickens
29	Yes	30-39	Husband and children	SABE employee	Home	Chickens, cows, pigs
30	Yes	20-29	Husband and children	Agriculture	Home and livestock	Chickens, goats, cattle
31	Yes	40-49	Husband and children	Crops	Home	Chickens, pigs
32	Yes	20-29	Children	Agriculture	Shop, home, livestock	Chickens, pigs, cattle
33	No	<19	Parents			Cattle
34	No	40-49	Husband and children	Agriculture	Home	Chickens

2. **Study questionnaire for face to face interviews.**

Background questions:

1- How old are you?
2- What is your educational background?
3- Who do you live with?
4 – How many people are in your family?
5 – Do all your family live in the same area of the community?
6 – In your family, who goes to school? How old will they be when they leave?
7 –What is the main source of income in your household?
8- Do you spend your day to day life working at home or do you have your own job?
9 – How big is your community?
10 – How far do you travel to: Work? School? Hospital/doctors?
11- How is your community run? E.g. community leaders etc
12 – What are the daily roles of men, women and children in your community?

How do equids contribute to the livelihoods of women in Chimaltenango?

1. Do you or your family own a donkey/horse/mule? If so, how many? What other animals other than equids do you own?
2. Rank ALL your animals in order of importance. Why have you put them in this order?
3. What is your donkey/horse/mule used for?

Income

- How do your donkeys/mules/horses make money?
- Who decides what kind of work your donkeys/mules/horses do to earn money?
- Who decides how the money earned by working donkeys/mules/horses is spent?
- How is the money earned by equids used?
- Do people with donkeys/mules/horses earn more than people without?

Savings (time, labour)

- How do your working equids help with household chores?
- How does having an equid to help with household chores impact your life?
- Do you have more or less chores to do because your family has a horse/ mule/ donkey?

Loans

- Does owning a donkey/horse/mule mean that you can take out a loan or get credit?
- Have you ever used a donkey/horse/mule to take out a loan or get credit?

Social

- Does your donkey/horse/mule help you to join social groups or spend time with friends?
- Do you let other people borrow your donkey/horse/mule? Who? Why?
- Who decides whether they can be lent and who to lend them to?
- If you have a donkey/mule/horse does that make you more or less respected than other women who don't have donkeys/mules/horses?

Education

- Does your donkey/horse/mule affect your ability to access school? How?

Food

- How does your donkey/horse/mule affect food production?
- How does your donkey/horse/mule support other livestock?

The impact of poor health

- What impact does a sick or lame donkey/mule/horse has on your lives?

On income?
Your ability to do household chores?
Social benefits?

3. What happens if your donkey/horse/mule dies?

Does it affect income?
Your ability to do household chores?
Social benefits?

What roles do women in Chimaltenango have to care for working equids?

1. What role do you have in caring for the horse/donkey/mule?
2. Do you use the horse/donkey/mule yourself or does somebody else?
3. Where do you go if your horse/donkey/mule is sick or lame?
4. Who decides how much to spend on treatment?
5. Who makes the decision about how the donkeys/mules/horses are looked after when they are sick?
6. Do you use veterinary treatments or local medicinal treatments? Why?

What level of knowledge and skills do women currently have to undertake these tasks?

1. Who originally taught you to care for your horse/donkey/mule?
2. What aspects of working with or looking after donkeys/mules/horses don't you do or can't you do? Why not?
3. Do you feel that you have as much knowledge on caring for donkeys/horses/mules as you would like?
4. How has your personal level of knowledge changed since SABE and World Horse Welfare set up their project?

What opportunities do women have to acquire new equid husbandry knowledge and skills?

1. Where do you get new knowledge and skills about looking after donkeys/mules/horses?
2. What training or opportunities are there to learn about donkeys/mules/horses? Who goes to these?
3. If they are available who decides whether you go? Why?

Do women in Chimaltenango find training in equid welfare helpful? If so, are there specific parts of equid care on which they would like to build their skills?

1. Do you find the opportunities to learn about how to care for horses/donkeys/mules, helpful?
2. If you had to make any improvements to current training what would they be?
3. What other types of training or education about donkeys/mules/horses would you like to have if they were more available/accessible?
4. What area of equid care would you like to build your skills? Why?
